# Comparative study of aromatic compounds in fruit wines from raspberry, strawberry, and mulberry in central Shaanxi area

**DOI:** 10.3402/fnr.v59.29290

**Published:** 2015-11-27

**Authors:** Yiming Feng, Min Liu, Yanan Ouyang, Xianfang Zhao, Yanlun Ju, Yulin Fang

**Affiliations:** 1 College of Enology, Northwest A&F University, Yangling, China; 2 Shaanxi Engineering Research Center for Viti-Viniculture, Yangling, China

**Keywords:** aroma compounds, solid-phase microextraction, strawberry, raspberry, mulberry

## Abstract

**Background:**

Although grape wines have firmly dominated the production and consumption markets of fruit wines, raspberry, strawberry, and mulberry have been utilized to make wines because of their joyful aroma and high contents of polyphenolic phytochemicals and essential fatty acids. However, little is known about aromatic compounds of the wines produced from these three fruits.

**Methods:**

The aromatic composition of fruit wines produced from raspberry, strawberry, mulberry, and red grape was analyzed by GC-MS. Odor activity values (OAVs) and relative odor contributions (ROCs) were used to estimate the sensory contribution of the aromatic compounds to the overall flavor of the wines.

**Results:**

In strawberry, raspberry, and mulberry wines, 27, 30, and 31 odorants were detected, respectively. Alcohols formed the most abundant group, followed by esters and acids. The grape wine contained a wider variety (16 types) of alcohols, and 4-methyl-2-pentanol and 2,3-butanediol were not present in the three fruit wines. The quantity of esters in raspberry (1.54%) and mulberry wines (2.08%) were higher than those of strawberry wine (0.78%), and mulberry wine contained more types of esters. There were no significant differences of acids between the three fruit wines and the control wine. In addition, 2-heptanone, 2-octanone, 2-nonanone, and 2-undecanone were unique to raspberry wine, and nonanal was present only in mulberry wine. The indistinguishable aroma of the three fruit wines was attributed to the dominance of fruity and floral odor components derived from ethyl esters of fatty acids and their contributions to the global aroma of the three fruit wines.

**Conclusion:**

The present study demonstrated that there were significant differences in the volatile components of fruit wines made from raspberry, strawberry, and mulberry. The aroma compounds were more abundant in the raspberry and mulberry wines than in the strawberry wine, but the quality of strawberry wine was superior to raspberry and mulberry wines.

Although grape wines have firmly dominated the production and consumption markets of fruit wines, several other fruits with the potential for use in wine production have been utilized to make wines by an increasing number of researchers and producers aiming to satisfy the desire for diversity in wine product consumption. Research on the nutritional value of suitable fruits other than grapes has been conducted continually (1).

Raspberries, *Rubus idaeus* L., have a high content of polyphenolic phytochemicals, particularly flavonoids such as anthocyanin pigments, which give raspberries their characteristic color. The phytochemicals in raspberries may present significant antioxidant activity and may act as a protectant against biological oxidative stress in mammalian cells (2). Phenolic acids, such as *p*-coumaric, caffeic, ferulic, and ellagic acids, are commonly found in raspberries (3). Red raspberries, in particular, are known to demonstrate strong antioxidant capacity, mainly as a result of their high levels of anthocyanins and other phenolic compounds (2, 4–6).

The strawberry is a classic example of a sought-after quality fruit (7). Joyful aroma is an important character of high-quality strawberries. The intensity of the fragrance is the most important index in the evaluation of strawberry cultivars (8). The perishability and inherently short life of the fruit can result in rapid changes in the volatile compound profile (9).

The mulberry belongs to the genus *Morus* of the family Moraceae. There are 24 species of *Morus* and one subspecies, with at least 100 known varieties (10, 11). Mulberries contain essential fatty acids that humans cannot synthesize, and which must be obtained through diet. Essential fatty acids are long-chain polyunsaturated fatty acids derived from linolenic, linoleic, and oleic acids, and they are necessary for the formation of healthy cell membranes, the proper development and functioning of the brain and nervous system, and for the production of hormone-like substances called eicosanoids (thromboxanes, leukotrienes, and prostaglandins). These chemicals regulate numerous body functions, including blood pressure, blood viscosity, and immune and inflammatory responses (12).

The characteristic flavor of a fruit is due to the production of specific volatile flavor compounds in conjunction with a complex interaction of sugars, organic acids, and phenolics (13–16). Alcoholic fermentation leads to a series of byproducts in addition to ethanol. These include carbonyl compounds, alcohols, esters, acids, and acetals, all of which influence the quality of the final beverage. The composition and concentration of the byproducts can vary widely from a few ng/L to hundreds of mg/L (17).

Therefore, it is worthwhile to produce wines from these three fruits in order to preserve the quality of their hygienic function and for taste titillation. The fraction of current studies that focus on the aromas of raspberry, strawberry, and mulberry wine compositions is still small. It is possible to assess and differentiate wine quality according to the aromatic composition, given that the volatile components of wine represent a group of compounds with highly distinguishing characteristics, and can be determined by objective methods (18).

In this article, we compared the aromatic compounds of the three fruit wines from raspberry, strawberry, and mulberry, and attempted to determine the difference between the three fruit wines and Cabernet Sauvignon red wine in order to enhance the aroma characteristics of the three fruit wines. Attempts were also made to elucidate the origin of the aroma distinction of the wines.

## Materials and methods

### Winemaking

Raspberry, strawberry, and mulberry fruits (planted in 2012, fruited in 2014) were harvested from Fengxiang County Fruiter Experiment Centre, Shanxi Province, China. The fruit trees grow well with routine management. The berries were handpicked.

**Fig. 1:**
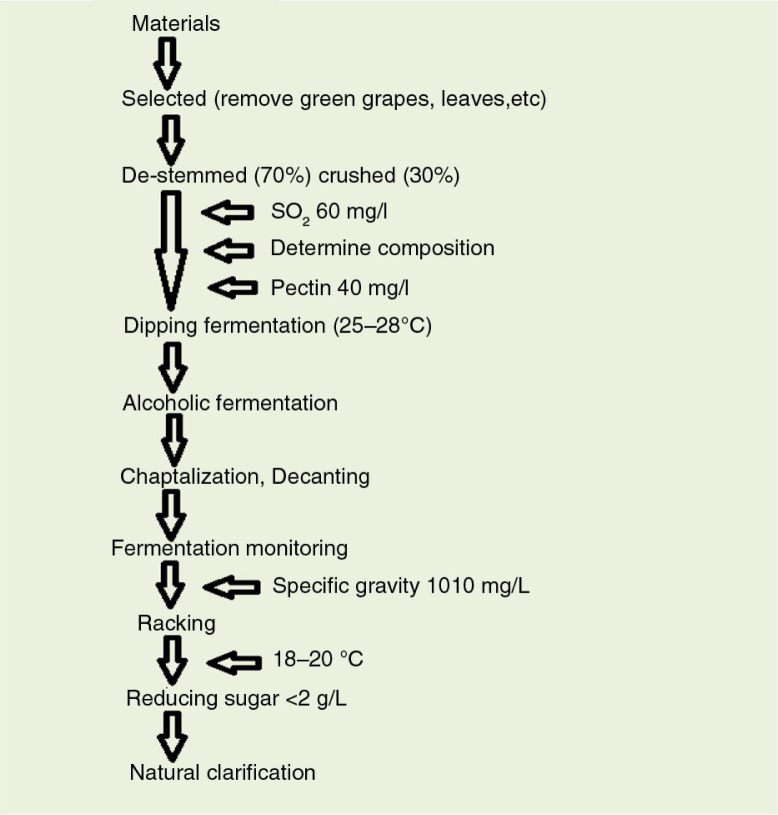
Winemaking procedure scheme following the traditional methods for red and fruit wines.

Briefly, the fruits were crushed, and sulfur dioxide (60 mg/L) and pectin were added to the musts with enzymolysis at 35°C for 2 h. Subsequently, 200 mg/L of activated dry yeast (CY3079) was added to the musts. Alcoholic fermentation was carried out at 18°C in 20,000 mL volumetric glass jars in accord with the dry red wine production process. The wines were not processed by fining filtration and deacidification treatment after natural clarification (19–21). Control samples of Cabernet Sauvignon (*Vitis vinifera* L. cv. Cabernet Sauvignon) single variety grapes were harvested from Manasi, Sinkiang Province, China, and vinificated in the Suntime Wine Company. A diagram of the process is shown in Fig. 1.

### Solid-phase microextraction

Aromatic compounds of the fruit wine samples were extracted by solid-phase microextraction (SPME) and analyzed by gas chromatography/mass spectrometry (GC-MS) as described by Zhang et al. (22). Five milliliters of wine sample and 1 g NaCl were placed in a 15-mL sample vial. The vial was tightly capped with a PTFE (polytetrafluoroethylene)-silicon septum and heated at 40°C for 30 min on a heating platform with agitation at 400 rpm. The SPME (50/30-µm DVB/Carboxen/PDMS, Supelco, Bellefonte, PA), preconditioned according to the manufacturer's instruction, was then inserted into the headspace, where extraction was allowed to occur for 30 min with continued heating and agitation by a magnetic stirrer. The fiber was subsequently desorbed in the GC injector for 25 min.

### GC-MS analysis

Compound profiling was performed using an Agilent 6890 GC-MS system equipped with an Agilent 5975 mass spectrometer and an HP-INNOWAX capillary column (60 m×0.25 mm) with 0.25-µm film thickness (J & W Scientific, Folsom, CA). Helium at a flow rate of 1 mL/min was used as the carrier gas. Samples were injected in the splitless mode by placing the SPME fiber at the GC inlet for 25 min. The oven was held at an initial temperature of 50°C for 1 min, and subsequently, raised to 220°C at a rate of 3°C/min and maintained at 220°C for 5 min. Mass spectra were recorded in the electron impact mode (MS/EI) at 70 eV in the range m/z 20 to 450 U. The mass spectrophotometer was operated in the selective ion mode under autotune conditions and the area of each peak was determined using ChemStation software (Agilent Technologies) (22).

Mass spectral data of each component were automatically retrieved by the NIST05 standard library, followed by checking and confirmation of the computer retrieval results against reference relative standard spectrograms. Based on the standard samples, calibration curves were derived for calculating each group of concentrations.

Odor activity values (OAVs) and relative odor contributions (ROCs) were used to estimate the sensory contribution of the aromatic compounds to the overall flavor of the wines. OAVs were calculated by dividing the mean concentration of an aromatic compound by its odor threshold value (22, 23). The ROC of each aroma compound was calculated as the ratio of the OAV of the respective compound to the total OAV of each wine (24). The OAVs of aromatic compounds in the three fruit wines were calculated by dividing the mean concentration of an aromatic compound by its odor threshold value.

### Statistical analysis

A one-way ANOVA test was used to evaluate the differences in the aromatic composition of the three fruit wines studied. Significant difference was calculated at the 0.05 level. SPSS version 18.0 Statistical Package for Windows was used for all statistical analyses.

## Results and discussion

### Physicochemical characteristics of musts and wines

Table 1 shows some of the physicochemical characteristics of the musts and wines from raspberry, strawberry, mulberry, and Cabernet Sauvignon. The results show that the sugar, titratable acidity, and pH of the musts were quite similar in the three non-grape varieties. Furthermore, no significant differences were observed in the titratable acidity, volatile acidity, pH, reducing sugars, or ethanol concentration of the young fruit wines made from the three kinds of berries after fermentation. However, due to the difference in the total sugar of the three berry wines versus Cabernet Sauvignon, the variation in the ethanol content of the wine was discriminating.

**Table 1:** General composition of musts and wines

		Total sugar (g/L)	Total acidity (g/L)	pH	Volatile acidity (g/L)	Reducing sugar (g/L)	Ethanol (%, v/v)
Raspberry	Must	120	8.8	3	–	–	–
	Wine	–	3.968	2.9	0.3203	1.82	6.09
Strawberry	Must	118	9.2	3.4	–	–	–
	Wine	–	3.273	2.8	0.3002	1.627	5.99
Mulberry	Must	120	9	3.2	–	–	–
	Wine	–	3.95	3	0.3434	1.653	6.1
Cabernet Sauvignon	Must	190	9.43	3.41	–	–	–
	Wine	–	6.8	2.8	0.24	2.1	11.8

### Composition of aroma

Three typical total ion chromatograms were generated for raspberry, strawberry, and mulberry wines using HS-SPME coupled with GC-MS. The key aromatic compounds of the three fruit wines were identified and grouped into alcohols, esters, acids, aldehydes, and ketones and compared with Cabernet Sauvignon (Table 2).

**Table 2:** Comparison of volatile components found in the raspberry, strawberry, mulberry fruit wines, and Cabernet Sauvignon young red wine

		Raspberry		Strawberry		Mulberry		Cabernet Sauvignon	
					
Compounds	Aroma description	Concentration (µg/L)	Relative content (%)	Concentration (µg/L)	Relative content (%)	Concentration (µg/L)	Relative content (%)	Concentration (µg/L)	Relative content (%)
**Alcohols**	**Number**	**15**		**13**		**13**		**16**	
1-Propanol	Bouquet, ripe fruity odor	–		3196.09	0.015	–		3058.80	0.33
2-Methyl-1-propanol	Bitter apricot seed odor	152295.13	2.12	778598.38	3.60	480445.44	5.50	nq	2.29
1-Butanol	Intoxicating aroma, alcoholic odor	–		19404.83	0.090	–		3617.84	0.16
2-Octanol	Unpleasant aromatic plant odor	nq		–		–		–	–
2-Hexanol	Coconut odor	–		nq		nq		nq	0.14
3-Methyl-1-butanol	Cheese odor	3745008.5	52.22	20568833.69	95.24	7885746.25	90.21	nq	71.88
1-Pentanol	Bouquet, astringent	20003.36	0.28	–		–		–	–
4-Methyl-1-pentanol	–	62101.11	0.86	58311.91	0.27	55354.75	0.63	7724.32	0.013
2-Heptanol	Brass odor, lemon odor	2456126.94	34.25	–		–		–	–
(*S*)-(+)-3-Methyl-1-pentanol	–	–		nq		–		nq	0.052
1-Hexanol	Light branches, leafy and fruity odor	5036.00	0.07	25214.18	0.12	51726.92	0.59	4468.30	1.35
(*Z*)-3-Hexen-1-ol	Strong fruity odor, light leafiness and green grass odor	–		–		10726.92	0.12	–	–
1-Octen-3-ol	–	–		–		8249.47	0.094	–	–
1-Heptanol	Bouquet plant odor, grape odor	20101.75	0.28	6774.95	0.031	36784.86	0.42	nq	0.01
(*S*)-3-Ethyl-4-methylpentanol	–	nq		–		–		nq	0.10
2-Nonanol	Strong fruity odor, rose odor	350953.11	4.89	–		–		–	–
3,7-Dimethyl-1,6-octadien-3-ol	–	nq		–		73191.05	0.84	–	–
1-Octanol	Fresh oranges and rose odor	286530.39	4.00	116603.70	0.54	122485.06	1.40	37210.27	0.18
[*R*-(R*,R*)]-2,3-butanediol	Rubber-like chemical odor	–		nq		–		nq	0.58
1-Nonanol	–	nq		nq		nq		nq	0.11
(*Z*)-3-Nonen-1-ol		–		–		nq		–	–
3,7-Dimethyl-(*R*)-6-octen-1-ol	–	47406.78	0.66	–		–		–	–
Phenylethanol	Sweet rose odor	25569.68	0.35	19528.95	0.090	16465.82	0.19	nq	3.38
4-Methyl-2-pentanol	–	–	–	–	–	–	–	nq	0.051
2,3-Butanediol	Rubber-like chemical odor	–	–	–	–	–	–	59.825	–
Subtotal (µg/L)		7171132.75		21596466.7		8741176.55		56139.355	
Subtotal (%)			96.245		98.85		96.34		80.63
**Esters**	**Number**	**9**		**9**		**12**		**7**	
Ethyl acetate	Fruity odor, ester odor	65825.675	57.32	37837.79	22.19	31408.88	16.62	8094.22	1.71
2-Methyl-ethyl butyrate	Sweet, fruity odor	nq		–		–		–	–
3-Methyl-ethyl butyrate	Fruity odor, fennel odor	8191.68	7.13	34543.32	20.26	18690.32	18.89	–	–
Hexyl acetate	Pleasant fruity odor, pear odor	19308.80	16.81	37618.19	22.07	48181.42	25.49	–	–
Ethyl hexanoate	Green apple odor, fruity odor	–		5324.33	3.01	11714.65	6.20	nq	2.38
Ethyl enanthate	Fresh fruit odor	–		–		1530.94	0.81	–	–
2-Hydroxy-(*S*)-ethyl propionate	–	1192.33	1.04	1611.56	0.94	–		nq	0.13
Methyl octanoate	Strong orangey odor	–		–		45313.11	23.97	214082.2	0.90
Ethyl octanoate	Fruity odor	5196.19	4.52	42821.22	25.12	23764.31	12.57	nq	5.98
Ethyl decanoate	Fruity odor	1245.2135	1.08	2106.66	1.24	1161.21	0.61	nq	1.07
Ethyl benzoate	–	–		–		ns			
Diethyl succinate	–	4060.39	3.54	1204.05	0.71	1463.82	0.77	nq	0.19
2-Hydroxy-methyl benzoate,	–	–		–		nq	–	–	–
Phenylethyl acetate	Floral odor	9824.48	8.55	7412.36	4.35	5804.04	3.07	–	–
Subtotal (µg/L)		114844.76		170479.47		189032.71		222176.42	
Subtotal (%)			1.54		0.78		2.08		12.36
**Acids**	**Number**	**2**		**4**		**4**		**4**	
Acetic acid	Strong smell	163633.52	99.06	67076.35	85.38	69684.97	70.42	18634.99	0.48
2-Methyl-propanoic acid	–	–		8790.76	11.19	12213.80	12.34	nq	0.1
Hexanoic acid	Unpleasant copra oil odor	–		nq		7974.39	8.06	435.27	0.14
Octanoic acid	Light fruity acid odor	1553.38	0.94	2698.09	3.43	9086.34	9.18	516.42	0.28
Subtotal (µg/L)		165186.9022		78565.20		98959.50			
Subtotal (%)			2.22		0.36		1.09		1.10
**Aldehydes and ketones**	**Number**	**4**		**1**		**2**		**0**	
2-Heptanone	Acetone, floral, and geranium odor	nq		–		–		–	–
2-Octanone	Floral odor, green fruit odor	nq		–		–		–	–
2-Nonanone	Special plant odor, rose odor, tea odor	nq		–		–		–	–
Nonanal	Rose odor	–		–		24156.61	55.48	–	–
Benzaldehyde	–	–		1225.07	1.00	19387.46	44.52	–	–
2-Undecanone	Peach odor, geranium odor	nq						–	–
Subtotal (µg/L)				1225.07		43544.07			0
Subtotal (%)					0.0056		0.48		

The data were mean values of triplicate samples (maximum SD:±10%). −=not detected; nq = detected without standard substance.

#### Alcohols

Alcohols are formed from the degradation of amino acids, carbohydrates, and lipids (25, 26). Among the tested parameters, the alcoholic degree was the enological parameter that had the greatest effect on the accumulation of volatile compounds in the wines (27). Quantitatively, alcohols formed the most abundant group in the aromatic components of the three fruit wines, constituting 96.24 to 98.85% of the total aroma content of the wine, the percentage of alcohol in the aromatic components of the control Cabernet Sauvignon wine was 80.6325%, followed by esters and acids. This result differs from that of Zhang et al. (22) in which it was reported that acids formed the most abundant group of the aromatic compounds in wines.

Alcohols, with 25 compounds identified, represented the largest group in terms of the numbers of aromatic compounds identified. The composition of alcohols differed both qualitatively and quantitatively among the three fruit wines. 3-Methyl-1-butanol was the most abundant alcohol accounting for 52.22, 95.24, and 90.21% of the total higher alcohols in the raspberry, strawberry, and mulberry fruit wines studied, respectively, and the content of this alcohol was significantly higher in the strawberry and mulberry wines. There were no significant differences in the relative contents of the common alcohols of the three fruit wines. Compared to strawberry and mulberry wines, the alcohol profile of raspberry wine was more diverse, containing 15 types of alcohols compared to 13 in strawberry and mulberry wines. 1-Propanol and 1-butanol, which conferred an intoxicating aroma on strawberry wine, were absent in the wines made from raspberry and mulberry. 2-Heptanol, 2-heptanol-(*S*)-3-ethyl-4-methyl-pentanol, 2-nonanol, and (*R*)-3,7-dimethyl-6-octen-1-ol were the alcohols with the most distinguishing odors that were found only in raspberry wine. (*Z*)-3-Hexen-1-ol and 1-octen-3-ol were the alcohols found only in mulberry wine and conferred a rich, strong fruity odor, light leafiness and green grass odor. The major differences between the three fruit wines and the control wine were that the Cabernet Sauvignon wine contained a wider variety (16 types) of alcohols, and there were some differences in the relative content of the compounds. In addition, the 4-methyl-2-pentanol and 2,3-butanediol components of the Cabernet Sauvignon wine were not present in the three fruit wines.

#### Esters

There were also significant differences in the types and amounts of esters present in the three fruit wines. In general, the number and quantity of esters in raspberry (1.54%) and mulberry wines (2.08%) were higher than those of strawberry wine (0.78%), and mulberry wine contained more types of esters than the other wines. Although the ester content varied for the three fruit wines, ethyl acetate, ethyl butyrate, 3-methyl-hexyl acetate, and ethyl octanoate were the major esters found in the aromatic components of the three fruit wines and were more abundant in the raspberry and mulberry wines. Ethyl enanthate, methyl octanoate, ethyl benzoate, and 2-hydroxy-methyl benzoate were found only in mulberry wine, whereas 2-methyl-ethyl butyrate was uniquely present in raspberry wine. The ethyl esters of the medium-chain fatty acids (C6–C12) are produced during yeast fermentation by the reactions of ethanol and acyl-coenzyme A derivatives (28). These compounds appear mainly during the alcoholic fermentation phase (29). On the other hand, the formation of acetate esters is the result of the reaction between acetyl-CoA and alcohols (30). In the control Cabernet Sauvignon wine there were only seven types of esters but the total relative content was 12.36% higher than the total content of the three fruit wines.

#### Acids

The production of fatty acids has been reported to be dependent on the composition of the must as well as the fermentation conditions (31). In other words, the fatty acids in wines are mainly produced by fermentation (24). In general, the total acid content of the three fruit wines was low. There were only four types of acids identified, while only two types were present in raspberry wine. The formation of volatile organic acids during yeast fermentation is quantitatively low, but it cannot be neglected from the viewpoint of flavor (32). Acetic acid is produced during alcoholic and malolactic fermentation. At low levels, this compound enhances wine flavors; however, at high concentrations, it is detrimental to the taste of the wine whereby it confers a sour and thin taste to the wine (33). There were no significant differences between the three fruit wines and the control Cabernet Sauvignon wine in terms of the types and relative content of acids.

#### Aldehydes and Ketones

Carbonyl compounds mainly include aldehydes and ketones, most of which are produced by microbial activity. These compounds may confer a richer, more elegant and unique aroma to wine (24). The composition of aldehydes and ketones varied greatly among the three fruit wines. 2-Heptanone, 2-octanone, 2-nonanone, and 2-undecanone were unique to raspberry wine, in which benzaldehyde was absent. On the other hand, nonanal was present only in the aromatic components of mulberry wine. However, no aldehydes and ketones were present in the control Cabernet Sauvignon wine.

### Odor activity values

Table 3 shows the thresholds, OAVs and ROC of the compounds present the three fruit wines and control Cabernet Sauvignon wine. Eight types of compounds exceeded the threshold values and were present in all four wines: 2-methyl-1-propanol, 3-methyl-1-butanol, 1-octanol, ethyl acetate, 3-methyl-ethyl butyrate, hexyl acetate, ethyl decanoate, and phenylethyl acetate. The OAVs were similar (ethyl acetate, ethyl decanoate) or had a significant gap (2-methyl-1-propanol, 3-methyl-1-butanol, 1-octanol, 3-methyl-ethyl butyrate, hexyl acetate, phenylethyl acetate), but only 1 compound (1-octanol) was present in the control Cabernet Sauvignon wine which was at least several-fold lower than that of the three other fruit wines. Based on the ROC index it was found that the compounds that contributed to the general aroma of the fruit wines and the control Cabernet Sauvignon wine were significantly different. These included 3-methyl-ethyl butyrate, phenylethyl acetate and ethyl hexanoate, with fruity and floral odors that produced the fermentative aromas of the three fruit wines, whereas 1-octanol was dominant for the control Cabernet Sauvignon wine. At the same time, compounds that contributed to the global aroma of the fruit wines were more abundant in the strawberry wine. The global aroma of the three fruit wines was dominated by fermentative aromas that may be responsible for the indistinguishable aroma of the three fruit wines. On the other hand, the significant difference in the ROC value of the three fruit wines may be the source of the subtle distinctions, for example, the ROC of 3-methyl-ethyl butyrate in the raspberry, strawberry and mulberry wines was 2.84%, 43.48%, and 8.10%, respectively, and produced various levels of fruity odor and fennel odor, as was the case with phenylethyl acetate, ethyl hexanoate, and ethyl enanthate.

**Table 3:** Odor activity values (OAVs) and relative odor contribution (ROC) of the aroma compounds in raspberry, strawberry, mulberry fruit wines, and Cabernet Sauvignon young red wine

			Raspberry		Strawberry		Mulberry		Cabernet Sauvignon	
						
Compounds	Odor description	Threshold (µg/L)	OVAs	ROC (%)	OVAs	ROC (%)	OVAs	ROC (%)	OVAs	ROC (%)
1-Propanol	Bouquet, ripe fruity odor	50,000	–	–	0.064	0.0014	–	–	0.061	0.14
2-Methyl-1-propanol	Bitter apricot seed odor	40,000	3.81	0.024	19.46	0.44	12.01	0.094	–	–
1-Butanol	Intoxicating aroma, alcoholic odor	150,000	0.13	0.00081	–	–	–	–	0.024	0.055
3-Methyl-1-butanol	Cheese odor	40,000	93.62	0.58	514.22	11.65	197.14	1.54	–	–
1-Pentanol	Bouquet, astringent	80,000	0.25	0.0016	–	–	–	–	–	–
1-Hexanol	Light branches, leafy and fruity odor	5,200	0.97	0.0060	4.85	0.11	9.95	0.078	0.86	1.98
1-Octanol	Fresh oranges and rose odor	900	318.37	1.99	129.56	2.94	136.09	1.062	41.34	95.29
Ethyl acetate	Fruity odor, ester odor	17,000	3.87	0.024	2.22	0.050	1.85	0.014	0.48	1.11
3-Methyl-ethyl butyrate	Fruity odor, fennel odor	18	455.09	2.84	1919.07	43.48	1038.35	8.10	–	–
Hexyl acetate	Pleasant fruity odor, pear odor	1,500	12.87	0.080	25.08	0.57	32.12	0.25	–	–
Ethyl hexanoate	Green apple odor, fruity odor	14	–	–	380.31	8.62	836.76	6.53	–	–
Ethyl enanthate	Fresh fruit odor	400	–	–	–	–	3.83	0.030	–	–
Ethyl decanoate	Fruity odor	200	6.23	0.039	10.53	0.24	5.81	0.045	–	–
Phenylethyl acetate	Floral odor	0.65	15114.58	94.40	1403.63	31.80	8929.29	69.65	–	–
Acetic acid	Strong smell	200,000	0.82	0.0051	0.34	0.0077	0.35	0.0027	0.093	0.21
2-Methyl-propanoic acid	–	2,300	–	–	3.82	0.086	5.31	0.041	–	–
Hexanoic acid	Unpleasant copra oil odor	8,800	–	–	–	–	0.91	0.0071	0.49	1.13
Octanoic acid	Light fruity acid odor	15,000	0.10	0.00062	0.18	0.0041	0.61	0.0048	0.034	0.078
Nonanal	Rose odor	15	–	–	–	–	1610.44	12.56	–	–

**Fig. 2:**
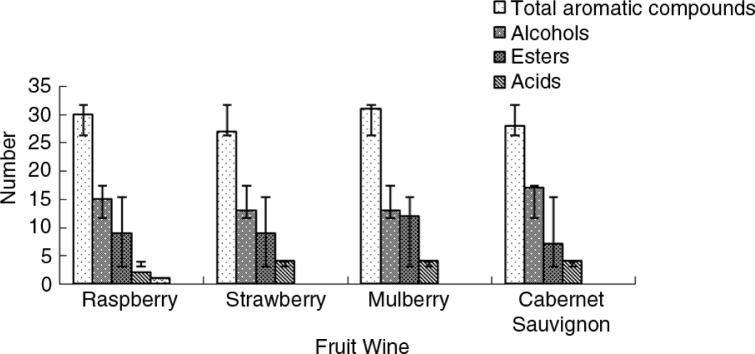
Variation of aromatic compounds in the three fruit wines.

General analysis of the variation in the number and content of aromatic compounds in the three fruit wines (Fig. 2) shows that the compounds that were significantly different in the various wines were esters, which was the main contributor to the difference in the sum of aromatic compounds. In general, the aromatic compounds were more abundant and the relative content of esters, aldehydes, and ketones was higher in the raspberry and mulberry wines than in the strawberry wine. On the other hand, a greater diversity of alcohols was present in the strawberry wine. Wine aroma is dominated by ethanol, but the distinctive characteristics are imparted by a large number of compounds that are present in small quantities. Typical compounds include higher alcohols, terpene alcohols, esters, phenolic and organic acids, ketones, and aldehydes. All of these compounds are present in small concentrations, ranging from 10^−1^ to 10^−10^ g kg^−1^ (34). In this study, the number and content of esters, acids, ketones and aldehydes, which constitute the aromatic compounds present in small quantities, were significantly different for the various wines. Based on the considerations presented above, the subtle distinctions in the three fruit wines are potentially mainly governed by the differences in the relative and total contents the aromatic compounds.

Aroma compounds play an important role in determining the quality of wine because these compounds elicit a sensory response (35). Normally, wine aroma is produced by a large number of volatile compounds and a specific ratio and or a combination of such compounds (36). The three fruit wines were evaluated by sensory descriptive analysis to obtain the aromatic descriptors. Descriptive analysis revealed that the three fruit wines were characterized with aroma descriptors belonging to seven groups: vegetal, floral, fruity, chemical, toast, nut, and metal odor.

Fang and coworkers found that ca. 33 kinds of volatile components were found in raw raspberry wine, and the alcohol compounds that were formed primarily during fermentation were the main components of the ultimate aroma composition. For instance, phenylethanol gave the raspberry wine a basic and abundant aroma character comprising rose, violet, jasmine, spicy/mint, anise, clove, fruity aroma, and so on (21). Some studies have reported that the compounds that contribute mainly to the flavor were fruity aromas that were primarily produced by alcohol and ester compounds in strawberry wine (9, 22, 37, 38). Chen and coworkers analyzed the aroma components of mulberries from different varieties, and found that the aroma components mainly included higher fatty acids, fatty acid esters, fatty alcohols, aromatic alcohols, acetaldehyde, aliphatic ketones, etc. It was also found that the relative content of higher fatty acids, which are important precursors to aroma development, was very high. Aldehyde, nonanal, hexanol, 3-methyl-butanol, 2,3-butanediol, phenylethanol, and 3-hydroxy-2-butanone were the main aroma constituents of the mulberry fruit and gave the mulberry fruity, floral, and green aromas (39). Studies on the influence of fermentation using different yeasts on the aroma components of mulberry wine indicated the presence of the same aroma components, including 3-methyl-butanol, 2,3-butanediol with fruity and floral aromas, in the mulberry wines fermented by different yeasts (40).

**Fig. 3:**
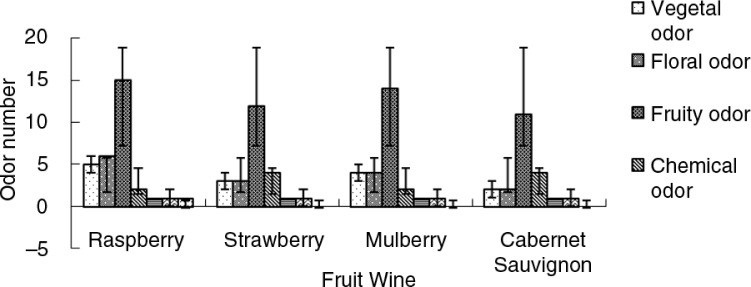
Variation of aroma descriptor groups.

The general overview of the analytical results (Fig. 3) indicates that qualitatively, the primary aroma descriptors that governed the flavor of the three fruit wines were fruity, floral, and vegetal aromas, compared with reports by Mar Vilanova et al. (35) that the compounds that mostly contributed to the flavor of Spanish Albariño wines were fruity and floral aromas. The compounds that mainly contributed to the flavor of Cabernet Sauvignon wine were fruity (1-propanol, 1-octanol, ethyl acetate, ethyl hexanoate, ethyl octanoate, ethyl decanoate, and octanoic acid) and chemical (ethyl alcohol, 1-butanol, 2,3-butanediol, and hexanoic acid) aromas. Nikfardjam and Maier reported that apple juices that were not made from concentrate were mainly characterized by flavor compounds responsible for fruity, ripe, and sweet aroma impressions, such as 1-butanol, 2-methyl-1-butanol, ethylbutyrate, and ethyl-2-methylbutyrate. In contrast, apple juices made from concentrate were dominated by acetaldehyde, (*E*)-2-hexenal, 3-methyl-1-butanol, ethyl acetate, and hexanal, which are mainly responsible for sensory impressions, such as green, fresh, and estery. Fig. 3 also shows that the most significantly different aromas in the three fruit wines studied are fruity aromas followed by floral and vegetal aromas. In other words, the compounds that contributed principally to the flavor were in keeping with the significantly different aromas (41).

## Conclusions

The present study demonstrated that there were significant differences in the volatile components of fruit wines made from raspberry, strawberry, and mulberry. The aroma compounds were more abundant in the raspberry and mulberry wines than in the strawberry wine, but the quality of strawberry wine was superior to raspberry and mulberry wines. The indistinguishable aroma of the three fruit wines was probably due to the dominance of fruity and floral odor components derived from ethyl esters of fatty acids and their contribution to the global aroma of the three fruit wines.
